# Monocyte at diagnosis as a prognosis biomarker in tuberculosis patients with anemia

**DOI:** 10.3389/fmed.2023.1141949

**Published:** 2023-06-07

**Authors:** Mengxing Luo, Xin Zou, Qibing Zeng, Yaxing Wu, Hua Yang, Lianhua Qin, Ruijuan Zheng, Fangyou Yu, Yang Hu, Zhonghua Liu

**Affiliations:** ^1^Shanghai Key Laboratory of Tuberculosis, Shanghai Pulmonary Hospital, Tongji University School of Medicine, Shanghai, China; ^2^The Key Laboratory of Environmental Pollution Monitoring and Disease Control, Ministry of Education, School of Public Health, Guizhou Medical University, Guiyang, China

**Keywords:** tuberculosis, anemia, monocyte, prognosis, biomarker

## Abstract

**Background:**

Anemia leads to a lower cure rate and poor prognosis in tuberculosis patients. Effective predictors for the prognosis of tuberculosis with anemia (A-TB) are urgently needed. Monocyte has been proven to be a prognostic biomarker of many lung diseases. Whether monocyte that the predominant innate immune cell as early defense against tuberculosis can predict A-TB is not known.

**Methods:**

Data for A-TB patients with initial treatment in Shanghai Pulmonary Hospital were retrospectively collected and analyzed. Logistics regression analysis was used to study the correlation between peripheral blood cells and treatment outcomes. The receiver operating characteristic (ROC) curve was used to determine the cut-off value. We estimated a 12-month prognosis using Kaplan–Meier techniques. The Cox proportional hazards model was used for the univariate and multivariate analyses to analyze the predictors of poor prognosis of A-TB.

**Results:**

Of 181 patients analyzed, 94 were cured and 87 non-cured. Logistic regression analysis identified monocyte as an independent immune-related risk factor for the prognosis of A-TB (OR: 7.881, 95% CI: 1.675–37.075, *P* = 0.009). The ROC curve analysis proved that the most discriminative cut-off value of monocyte was 0.535 × 10^9/L. K–M analysis demonstrated that the cumulative cure rates of A-TB were significantly higher in A-TB with monocyte < 0.535 × 10^9/L (69.62%) than that in those with monocyte ≥ 0.535 × 10^9/L (38.24%) (Log-rank, χ^2^ = 16.530, *P* < 0.0001). On univariate and multivariable analysis, monocyte was an independent predictor of poor prognosis in A-TB. Similarly, monocyte was also an independent predictor of poor pulmonary cavity closure in A-TB (HR: 3.614, 95% CI: 1.335–9.787, *P* = 0.011).

**Conclusion:**

In A-TB patients, elevated monocyte was associated with poor prognosis and poor cavity pulmonary closure. Monocyte may provide a simple and inexpensive prognostic biomarker in A-TB.

## Background

Tuberculosis (TB) is the number one cause of death from infectious diseases globally. All countries are affected by TB, but most cases were in adults (1). By 2022, China was the third highest TB burden country in the world (2). TB morbidity and mortality have probably declined for several years, but significant public health challenges remain, and major gaps in care and prevention remain (3). Anemia is a common complication of TB, ranging from 20% to 94% in TB patients (4, 5). Tuberculosis with anemia (A-TB) has increased sputum load and aggravated pulmonary infection. Anemia can lead to decreased cure rate and poor prognosis in TB patients (6). At present, there are few studies on the prognosis of A-TB, and no relevant reports on the predictors of A-TB prognosis have been found. Therefore, there is an urgent need for the identification of simple, clinician-friendly, cost-effective prognostic biomarkers for A-TB; these prognostic biomarkers could be used to stratify patients with similar clinical presentations at diagnosis who are at risk of more rapid disease progression (7). Identification of such biomarkers may aid in the development of more personalized treatment plans (7).

Peripheral blood cells have been used as a potential source of biomarkers to predict the prognosis of respiratory diseases (8–12). Mycobacterium tuberculosis (M.tb) invades the host, activates the immune responses and causes TB (13). Immune markers measured at the beginning of treatment can accurately predict the outcomes of TB treatment. Our previous study evaluated many factors of A-TB, immune factors such as complement C-4, C-reactive protein, and globulin α2, and immune cells such as lymphocytes, basophils, and monocyte. Some immune factors and immune cells suggested poor prognosis of A-TB (6). Monocyte is the predominant innate immune cell at the early stage of M.tb infection as the host defense against intracellular pathogens (14). In reports, monocyte has been proven to be a biomarker of poor prognosis in respiratory diseases such as idiopathic pulmonary fibrosis, lung adenocarcinoma, and interstitial lung disease (8, 9, 11, 15). Given that monocyte is a component of standard blood tests, this may represent a simple and inexpensive biomarker that warrants further investigation (9).

The short course chemotherapy scheme for M.tb recommended by WHO includes first-line drug for 4 months for sensitive bacteria and 6, 9, and 12 months for rifampicin-resistant TB (RR-TB), multidrug-resistant TB (MDR-TB), extensively drug-resistant TB (XDR-TB) (16, 17). In this study, a retrospective cohort study was conducted to investigate the value of monocytes in evaluating the 12-month prognosis of A-TB.

## Materials and methods

### Study population

**FIGURE 1:**
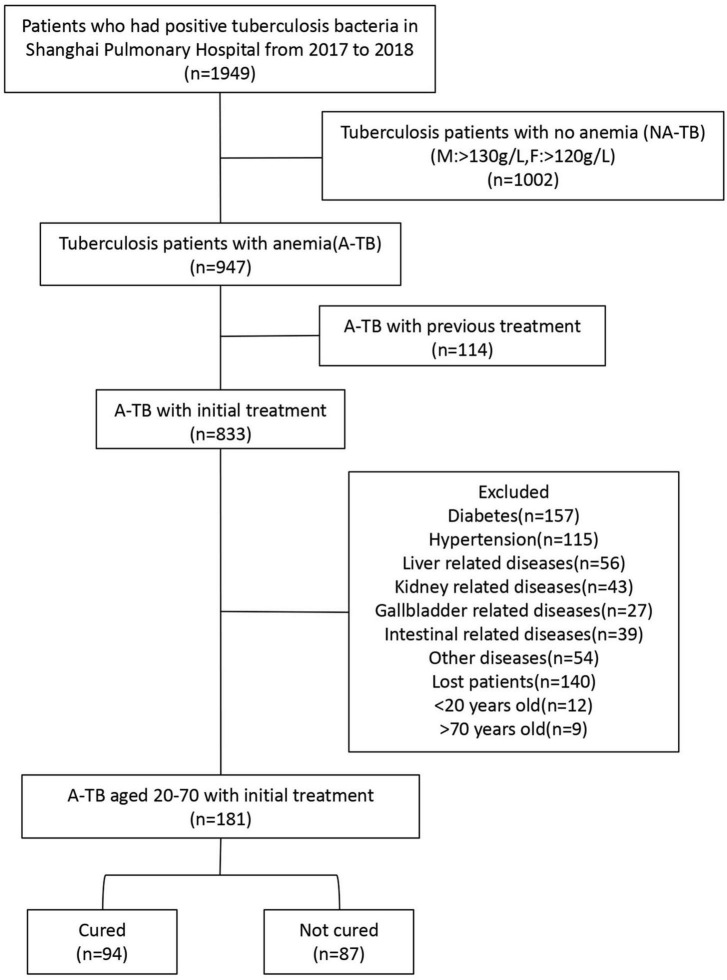
Study flow diagram including clinical treatment outcomes for patients within 12 months. Patients with recurrence, other complications and no post-treatment follow-up were excluded while patients aged 20–70 with complete data were included and grouped according to treatment outcome. Patients who had positive TB bacteria in Shanghai Pulmonary Hospital from 2017 to 2018.

In this cohort study, we retrospectively analyzed the cases of A-TB who were treated in Shanghai Pulmonary Hospital from January 2017 to December 2018. The inclusion criteria were A-TB who were sputum smear-positive between 20 and 70 years of age. The exclusion criteria were patients with previous TB history or treatment, patients without complete medical records, and patients with liver or kidney diseases, diabetes or other comorbidities (Figure 1).

### Data collection and definitions

All data of this study were obtained from the information processing database and electronic medical record system of Shanghai Pulmonary Hospital, including the baseline characteristics, clinical data at admission, imaging data, and treatment outcomes.

According to the WHO criteria, anemia was defined as a hemoglobin concentration below 120 g/L in women and below 130 g/L in men (18). According to definitions and reporting framework for TB published by WHO, at least two bacteriologically negative results after completion of treatment were considered as the cure, while the positive test for M.tb or no improvement in clinical symptoms was considered as treatment failure (19).

Computed tomography (CT) data were collected from 181 A-TB patients over the period of 12 months to examine cavities in diameter at diagnosis and cavity closure. CT images were read and interpreted by 2 experienced radiologists.

### Statistical analysis

Differences in demographic and clinical characteristics at baseline between groups were analyzed using Mann–Whitney U test or non-parametric test for continuous variables as well as the chi-squared test or Fisher’s exact test for categorical variables, as appropriate. Logistics regression analysis was used to study the correlation. Adjustment variables were selected as follows: sociodemographic variables of known clinical importance (sex and age) and variables with a *p*-value of < 0.05 in the univariate analysis. The receiver operating characteristic (ROC) curve analysis was used to determine the optimal cut-off values; values with maximum joint sensitivity and specificity were selected. We estimated a 12-month prognosis using Kaplan–Meier techniques. The Cox proportional hazards model was used for the univariate and multivariate analyses, and the hazard ratio (HR) and 95% confidence interval (95% CI) were calculated. All statistical analyses were performed using SPSS25.0 or GraphPad Prism 8, and *P*-values less than 0.05 were considered statistically significant.

### Ethics statement

This study was approved by the Ethics Committee of Shanghai Pulmonary Hospital, Tongji University School of Medicine (Shanghai, China) (K19-060Y). All participants gave verbal consent for the use of their clinical information for research purposes.

## Results

### Patient characteristics

From January 2017 to December 2018, a total of 1,949 sputum smear-positive TB patients were admitted to Shanghai Pulmonary Hospital, of whom 947 (48.59%) were anemic. A total of 766 patients were excluded due to history of TB or treatment (*N* = 114), presence of other comorbidities (*N* = 491), incomplete follow-up (*N* = 140), and age < 20 and > 70 years (*N* = 21). Finally, a total of 181 patients were enrolled in the study and grouped according to the outcome of TB treatment during the 12-month observation period, 94 (51.93%) patients were cured and 87 (48.07%) patients were not cured (Figure 1).

Table 1 summarizes the patient characteristics at baseline. There were 41 males (43.62%) in the cured group, and the median age was 31 years old; there were 37 males (42.53%) in the non-cured group, and the median age was 30 years. Among the 181 A-TB patients, there were 9 drug-resistant patients, 1 RR-TB and 1 MDR-TB in the cured group, and 5 RR-TB and 2 MDR-TB in the non-cured group (Supplementary Table 1). There was no significant difference between the cured group and the non-cured group in terms of cough, fever, and weight loss. The sputum smear grade and hemoglobin of the patients in the cured group at admission were not significantly different from those of the non-cured patients.

**TABLE 1:** baseline characteristics of A-TB.

Characteristics		Anemia		*P*-value
		Cured group (*n* = 94)	Non-cured group (*n* = 87)	
Sex	Male	41 (43.62)	37 (42.53)	0.883
	Female	53 (56.38)	50 (57.47)	
Age		31 (26–39.25)	30 (25–38)	0.499
Lose weight		10 (10.64)	8 (9.20)	0.746
Cough		62 (65.96)	65 (74.71)	0.198
Fever		22 (23.40)	28 (32.18)	0.187
Drug resistance		2 (2.13)	7 (8.05)	0.090
**Grade of sputum smear**				
1+		68 (72.34)	53 (60.92)	0.129
2+		15 (15.96)	12 (13.79)	
3+		8 (8.51)	17 (19.54)	
4+		3 (3.19)	5 (5.75)	
HGB, g/L		119.51 ± 16.71	115.28 ± 12.42	0.065

### Monocyte at baseline as an independent risk factor for the prognosis of A-TB

We analyzed peripheral blood cells of A-TB patients in the cured and non-cured group at the time of admission. As shown in Table 2, monocyte (*P* < 0.0001) and platelets (*P* = 0.016) of A-TB were significantly increased in the non-cured group, while red blood cell (RBC) (*P* = 0.011) and HCT (*P* = 0.042) were significantly decreased. However, there was no statistically significant difference between the cured and non-cured groups of non-anemic TB patients on these four factors (Supplementary Table 2). At the same time, there were no significant differences in the neutrophil, lymphocyte, eosinophil, and basophil between the cured group and the non-cured group of A-TB patients.

**TABLE 2:** Univariate analysis of peripheral blood cells in cured and non-cured groups.

Factor	Reference value	Anemia		*P*-value
		Cured group (*n* = 94)	Non-cured group (*n* = 87)	
MONO, 10^9/L	0.10–0.80	0.49 (0.38–0.64)	0.66 ± 0.24	< 0.0001
HGB, g/L	130.00–175.00	119.51 ± 16.71	115.28 ± 12.42	0.065
RBC, 10^12/L	4.30–5.80	4.37 (4.13–4.65)	4.19 (3.97–4.47)	0.011
NEUT, 10^9/L	2.00–7.70	3.67 (3.01–4.71)	4.02 (3.19–5.07)	0.078
LYM, 10^9/L	0.80–4.00	1.42 (1.16–1.86)	1.52 ± 0.45	0.488
EO, 10^9/L	0.05–0.30	0.09 (0.05–0.17)	0.09 (0.04–0.22)	0.577
BASO, 10^9/L	0.00–0.10	0.02 (0.01–0.03)	0.02 (0.01–0.03)	0.401
PLT, 10^9/L	125.00–350.00	272.94 ± 67.49	300.37 ± 83.91	0.016
HCT, L/L	0.40–0.50	0.37 ± 0.04	0.36 ± 0.03	0.042
MCV, fL	82.00–100.00	85.50 (81.00–88.00)	86.00 (82.00–89.00)	0.673
MCH, pg	27.00–34.00	28.00 (26.00–29.00)	28.00 (27.00–29.00)	0.755
MCHC, g/L	316.00–354.00	321.38 ± 11.64	320.70 ± 12.63	0.706
PCT, %	0.11–0.28	0.27 (0.25–0.31)	0.30 (0.25–0.34)	0.082
PDW, %	9.00–17.00	11.60 (10.70–12.70)	11.40 (10.48–12.13)	0.264
MPV, fL	9.00–13.00	10.15 (9.68–10.80)	10.00 (9.50–10.50)	0.133

Differences in monocyte, red blood cell, platelet and hematocrit were statistically significant in univariate analysis. *P* < 0.05 is considered statistically significant. MONO, monocyte; HGB, hemoglobin; RBC, red blood cell; NEUT, neutrophil; LYM, lymphocyte; EO, eosinophil; BASO, basophil; PLT, platelets; HCT, Hematocrit; MCV, mean corpuscular volume; MCHC, mean hemoglobin concentration; PCT, platelet crit; PDW, platelet distribution width; MPV, mean platelet volume.

We performed unconditional logistic regression analysis to further identify risk factors for the prognosis of A-TB. Among the four factors, monocyte, RBC, platelets, and HCT, monocyte at baseline was an independent immune-related risk factor for the prognosis of A-TB (OR: 8.097, 95% CI: 2.031–32.290, *P* = 0.003). After adjusting for confounding factors such as age, sex, and drug resistance, monocyte remained an independent immune-related risk factor for the prognosis of A-TB (OR: 7.881, 95% CI: 1.675–37.075, *P* = 0.009) (Table 3).

**TABLE 3:** Multivariate logistics analysis of the correlation between peripheral blood cells and poor prognosis.

Factor	OR (95% CI)	*P*^a^-value	OR1 (95% CI)	*P*^b^- value
MONO, 10^9/L	8.097 (2.031–32.290)	0.003	7.881 (1.675–37.075)	0.009
RBC, 10^12/L	0.518 (0.277–0.971)	0.040	0.664 (0.260–1.697)	0.393
PLT, 10^9/L	1.005 (1.001–1.009)	0.019	1.003 (0.999–1.008)	0.145
HCT, L/L	0.001 (0.000–0.810)	0.044	0.010 (0.000–1,299.234)	0.444

^a^In multivariate logistics analysis, monocyte was a risk factor for poor prognosis.

^b^After adjusting for age, sex and drug resistance, monocyte remained an independent risk factor for poor prognosis.

MONO, monocyte; RBC, red blood cell; PLT, platelets; HCT, hematocrit.

### Monocyte at baseline as a predictor of prognosis in A-TB

**FIGURE 2:**
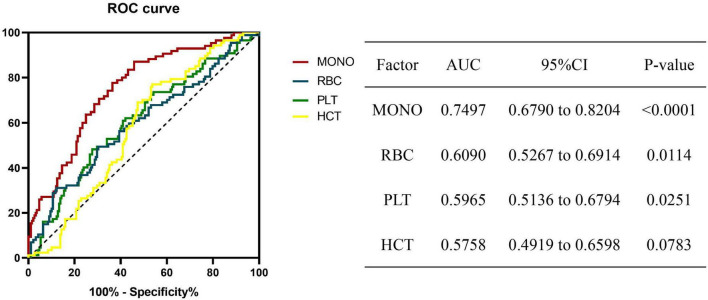
Receiver operating characteristic (ROC) analysis of significant biomarkers in univariate analysis in the prognosis for A-TB. ROC revealing a predictive value of monocyte as a biomarker of A-TB prognosis. MONO, monocyte; RBC, red blood cell; PLT, platelets; HCT, hematocrit.

The ROC curve results proved that monocyte at baseline could be used as a biomarker to predict the prognosis of A-TB. The most discriminative cut-off value of monocyte was 0.535 × 10^9/L, with an area under the curve (AUC) value of 0.7497 (95% CI: 0.6790 to 0.8204, *p* < 0.0001). RBC (cut off: 4.165 × 10^12/L, AUC: 0.6090, 95% CI: 0.5267–0.6914) and platelets (cut off: 294.5 × 10^9/L, AUC: 0.5965, 95% CI: 0.5136–0.6794) can also be used as biomarkers to predict the prognosis of A-TB (Figure 2).

**FIGURE 3:**
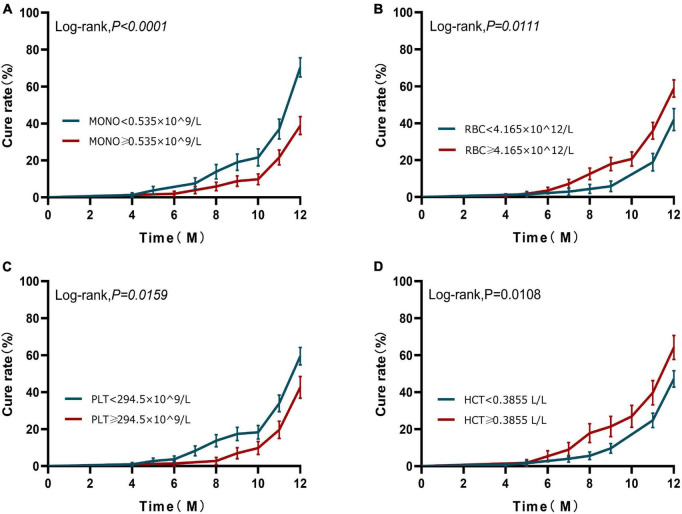
Kaplan–Meier curves of 12-month prognosis. **(A)** Analysis of cure status grouped according to monocyte at diagnosis during 12-month follow-up. **(B)** Analysis of cure status grouped according to RBC at diagnosis during 12-month follow-up. **(C)** Analysis of cure status grouped according to platelets at diagnosis during 12-month follow-up. **(D)** Analysis of cure status grouped according to HCT at diagnosis during 12-month follow-up. MONO, monocyte; RBC, red blood cell; PLT, platelets; HCT, hematocrit.

Based on the results of the ROC curve, we further grouped the patients according to the cut-off values of monocyte, RBC, and platelets, respectively. Kaplan–Meier analysis was used to investigate the effects of monocyte, RBC, and platelets on the cumulative cure rates of A-TB within 12 months. K–M analysis demonstrated that the cumulative cure rates of A-TB with monocyte < 0.535 × 10^9/L (69.62%) were significantly higher than that of patients with monocyte ≥ 0.535 × 10^9/L (38.24%) (Log-rank, χ^2^ = 16.530, *P* < 0.0001), as depicted in Figure 3A. The cure rates were significantly lower in the group with RBC < 4.165 × 10^12/L compared with the group with RBC ≥ 4.165 × 10^12/L (Log-rank, χ^2^ = 6.456, *P* = 0.0111) (Figure 3B). The cure rates were higher in the group with platelets < 294.5 × 10^9/L compared to the group with platelets ≥ 294.5 × 10^9/L (Log-rank, χ^2^ = 5.811, *P* = 0.0159) (Figure 3C). No statistical difference in ROC for HCT (Figure 3D).

Next, to examine if monocyte at baseline can work as an independent prognostic factor for A-TB, univariate and multivariate analysis were performed. In the univariate analysis, monocyte ≥ 0.535 × 10^9/L (*P* < 0.0001), RBC < 4.165 × 10^12/L (*P* = 0.007), and platelets < 294.5 × 10^9/L (*P* = 0.011) were significant predictors for poor prognosis of A-TB (Table 4). We then performed multivariate analyses in the Cox proportional hazard model adjusting for confounding factors such as age, sex, and drug resistance in order to further determine whether monocyte, RBC, and platelets were independent predictors for poor prognosis of A-TB. As shown in Table 4, monocyte ≥ 0.535 × 10^9/L [hazard ratio (HR) 2.100, 95% CI: 1.378–3.198, *P* = 0.001] was able to independently predict poor prognosis of A-TB.

**TABLE 4:** Univariate and multivariate Cox regression analysis of peripheral blood cells associated with prognosis of 181 patients with TB and anemia.

Factor	Group	Univariate analysis^a^		*P*-value	Multivariate analysis^b^		*P*-value
		Hazard ratio	95% CI		Hazard ratio	95% CI	
MONO, 10^9/L	<0.535	1.743	1.312–2.316	<0.0001	2.100	1.378–3.198	0.001
	≥0.535	0.521	0.363–0.746				
RBC, 10^12/L	<4.165	0.654	0.470–0.911	0.007	0.689	0.407–1.165	0.165
	≥4.165	1.515	1.128–2.034				
PLT, 10^9/L	<294.5	1.481	1.074–2.041	0.011	1.447	0.923–2.268	0.107
	≥294.5	0.676	0.502–0.909				
HCT, L/L	<0.3855	0.690	0.528–0.902	0.011	0.683	0.397–1.174	0.168
	≥ 0.3855	1.603	1.076–2.390				

^a^Univariate analysis, Cox proportional hazards regression.

^b^Multivariate analysis, Cox proportional hazards regression adjusting for age, sex, drug resistance, red blood cell, platelets and hematocrit.

CI, confidence interval; HR, hazard ratio; MONO, monocyte; RBC, red blood cell; PLT, platelets; HCT, hematocrit.

### Monocyte at baseline as a predictor of pulmonary cavity closure in A-TB

**FIGURE 4:**
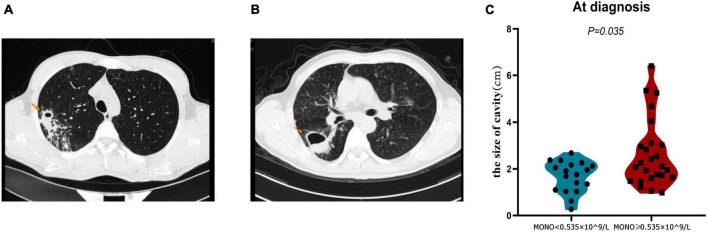
Comparison of cavity size at diagnosis of TB in monocyte < 0.535 × 10^9/L and monocyte ≥ 0.535 × 10^9/L patients with TB and anemia. **(A)** Pulmonary imaging data of a patient in monocyte < 0.535 × 10^9/L group. **(B)** Pulmonary imaging data of a patient in monocyte ≥ 0.535 × 10^9/L group. **(C)** The cavity size in monocyte ≥ 0.535 × 10^9/L group was significantly larger than that in monocyte < 0.535 × 10^9/L group. MONO, monocyte.

The closure of the pulmonary cavity in A-TB is an important indicator to judge the prognosis of patients (20). Therefore, we analyzed the cavity status of patients with monocyte < 0.535 × 10^9/L and monocyte ≥ 0.535 × 10^9/L in order to determine whether monocyte at baseline can also be used as a predictor of pulmonary cavity closure in A-TB. We analyzed the size of pulmonary cavities on admission in both groups, and patients with monocyte ≥ 0.535 × 10^9/L had relatively larger cavities compared with those with monocyte < 0.535 × 10^9/L (*P* = 0.035) (Figure 4).

**FIGURE 5:**
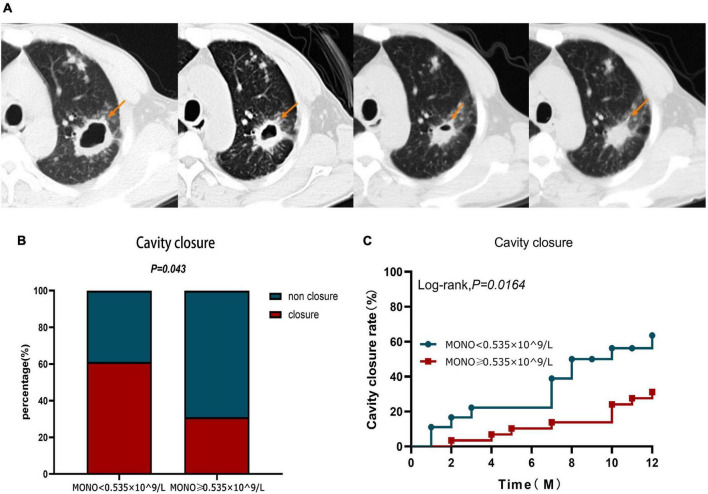
Cavity closure in patients with TB and anemia in monocyte < 0.535 × 10^9/L and monocyte ≥ 0.535 × 10^9/L group within 12 months. **(A)** In one patient, the size of the cavity changed over a 12-month period. **(B)** Difference in cavity closure ratio between monocyte < 0.535 × 10^9/L and monocyte ≥ 0.535 × 10^9/L group within 12 months. **(C)** Analysis of cavity closure grouped according to monocyte at diagnosis during 12-month follow-up. MONO, monocyte.

Observing the cavity closure of the two groups within 12 months (Figure 5A), the cavity closure rate in patients with monocyte < 0.535 × 10^9/L (61.11%) was significantly higher than that in patients with monocyte ≥ 0.535 × 10^9/L (31.03%) (*P* = 0.043) (Figure 5B). At the same time, the cavity closure rate estimated by the Kaplan–Meier method was significantly lower at 12 months when the initial monocyte level exceeded 0.535 × 10^9/L (Log-rank, χ^2^ = 5.759, *P* = 0.0164) (Figure 5C). Similar to the Cox proportional hazard model for the prognosis of A-TB, the multivariate Cox proportional hazard analysis was used to evaluate the validity of the index to identify the independent predictors of pulmonary cavity closure. The results demonstrated that only monocyte ≥ 0.535 × 10^9/L at baseline was an independent predictor of poor pulmonary cavity closure in A-TB (HR: 3.614, 95% CI: 1.335–9.787, *P* = 0.011) (Table 5).

**TABLE 5:** Univariate and multivariate Cox regression analysis of peripheral blood cells associated with cavity closure of 47 patients with TB and anemia.

Factor	Group	Univariate analysis^a^		*P*-value	Multivariate analysis^b^		*P*-value
		Hazard ratio	95% CI		Hazard ratio	95% CI	
MONO, 10^9/L	<0.535	1.969	1.022–3.794	0.043	3.614	1.335–9.787	0.011
	≥0.535	0.564	0.301–1.057				
RBC, 10^12/L	<4.165	0.794	0.39–1.615	0.514	0.885	0.275–2.845	0.837
	≥4.165	1.179	0.725–1.918				
PLT, 10^9/L	<294.5	1.322	0.625–2.796	0.449	1.076	0.401–2.889	0.885
	≥294.5	0.824	0.507–1.339				
HCT, L/L	<0.3855	0.871	0.439–1.726	0.696	1.107	0.355–3.452	0.861
	≥0.3855	1.113	0.640–1.936				

^a^Univariate analysis, Cox proportional hazards regression.

^b^Multivariate analysis, Cox proportional hazards regression adjusting for age, sex, drug resistance, RBC, platelets and HCT.

CI, confidence interval; HR, hazard ratio; MONO, monocyte; RBC, red blood cell; PLT, platelets; HCT, hematocrit.

## Discussion

Anemia is the most common comorbidity in TB (21). In this study, 48.59% (947/1,949) of the TB patients with sputum smear-positive had anemia. There is some evidence to suggest that anemia at the time of TB diagnosis is associated with an increased risk of death (22–24). Compared with non-anemic TB patients, the cure rate of TB patients with anemia had a 21.85% lower cure rate, significantly delayed positive conversion of sputum, and worse recovery from lung damage (cavity, effusion, etc.) was worse (6). Therefore, it is necessary to predict the prognosis of A-TB. The earlier the prognosis can be predicted, the earlier interventions can be taken to improve the cure rate, shorten the course of treatment, minimize damage, and promote recovery, striving to minimize the damage caused by TB to patients. Up to now, there are few studies on the prognosis of A-TB, while no biomarkers have been identified to predict the poor prognosis of A-TB.

In this cohort study, we retrospectively analyzed data from 181 A-TB patients. After adjusting for confounding factors such as age, sex, and drug resistance, analysis of peripheral blood cells such as neutrophils, lymphocytes, monocytes, and erythrocytes at diagnosis revealed that monocyte at baseline was an independent risk factor for poor prognosis of A-TB. Our data show that monocyte was significantly lower in cured A-TB patients than in non-cured patients. Multivariate analysis showed that monocyte was an independent predictor for the prognosis of A-TB; in other words, patients with monocyte < 0.535 × 10^9/L had a better prognosis than those with monocyte ≥ 0.535 × 10^9/L.

In recent years, monocyte has been increasingly studied as a prognostic biomarker for lung diseases, especially pulmonary fibrosis, pulmonary embolism, and lung tumors (8, 9, 11, 25). Research has demonstrated that a monocyte count of 0.60 to 0.95 × 10^9 cells/L was significantly associated with a higher 1-year risk of idiopathic pulmonary fibrosis (IPF) progression, hospitalization, and mortality versus patients with a monocyte count of < 0.60 × 10^9 cells/L (9). Monocyte is also a very strong independent predictor of mortality in patients diagnosed with pulmonary embolism (25). Monocyte contributes to the inflammatory process through their differentiation into macrophages or dendritic cells in the tissue microenvironment (26), so peripheral blood monocyte count can be used to predict the possibility of postoperative recurrence in pathological stage I lung adenocarcinoma (8). The results of this study showed that the cure rate of A-TB was significantly increased when monocyte was < 0.535 × 10^9/L, which was consistent with the conclusion that the prognosis of idiopathic pulmonary fibrosis, pulmonary embolism, and tumor was better when monocyte was lower in the above study. These results suggest that it is universal for monocyte to predict the prognosis of diseases, but whether monocyte can play a prognostic role through the same mechanism needs further study.

TB infection is known to induce systemic inflammation and lung damage (27). Monocytes are prototypical innate immune cells, they are critical effectors and regulators of inflammation and the innate immune response. Monocytes produce inflammatory cytokines and take up cells and toxic molecules; they can also differentiate into inflammatory dendritic cells (DCs) or macrophages during inflammation (28, 29). Numerous transcriptomic approaches have revealed that many subsets of monocytes such as CD64 + and CD163 + and haptoglobin (HP) are known to be directly related to the progression and activity of TB (30–32). Current research focusing on monocytes and their subsets in TB has found that CD16 monocyte is expanded in TB infection (33), while expansion of CD16 monocyte is reversed with anti-TB treatment (34). It was shown that DS-TB patients had an increased frequency of platelets and monocytes compared to LTB (35). Our data, in A-TB patients, showed a significant increase in the number of platelets and monocytes in the non-cured group compared to the cured group, and, the increase in the number of monocytes in the non-cured group and the recovery of the cavity were positively correlated, consistent with reports, suggesting that our study is consistent with an immune mechanism of the platelet/monocyte axis. Although, the study showed no change in the frequency of classical and non-classical monocytes in DS-TB patients (35). Our data, in non-anemic TB patients, the number of platelets and monocytes did not change in the non-cured patients compared to the cured group, suggesting that monocytes are only a prognostic risk factor in A-TB patients and a key to our detailed analysis of A-TB monocytes. Brahmbhatt et al. (36) found that when TB patients were grouped according to sputum smear conversion at week 8, higher monocyte was observed in the patients with non-converted sputum smears at diagnosis. Meanwhile, the monocyte decreased with increasing duration of treatment (36). The above results show that when the treatment is effective and M.tb is cleared, the monocytes decrease in TB patients. However, in a prospective multicenter cohort study, no significant difference was observed in the monocyte count between cured and non-cured TB patients before treatment (12). Consistent with our previous results, we retrospectively analyzed the treatment outcomes of TB patients over a 3-year period and found no significant difference in monocyte at diagnosis between the cured and non-cured groups (6). In our study, we also found no significant difference in the monocyte at diagnosis between the cured group and the non-cured group based on 12-month treatment outcomes in TB patients with no anemia. Current studies have indicated that monocyte at the time of TB diagnosis cannot predict the prognosis of TB. Nevertheless, multivariate logistic analysis confirmed that monocyte was an independent risk factor for the poor prognosis of A-TB. That means monocyte at diagnosis could only be used as a predictor of the prognosis of A-TB.

At present, there was no direct evidence of monocyte regulation of anemia, and only a few studies have reported the correlation between monocyte and anemia. Anemia of inflammation is one of the most common anemia (37). Chronic infectious diseases, especially TB infection, often lead to inflammatory anemia (38). The latest discovery shows that the host defense mechanism is superior to the homeostatic process such as erythropoiesis in response to infection, it is explained that anemia patients maintain host defense at the expense of erythrocyte production and erythrocyte survival (37). Therefore, chronic infection is one of the causes of inflammatory anemia. Monocytes recruited to the spleen generate macrophages under anemia stress and then develop into macrophages that form erythroblastic islands, thereby coordinating the production of new red blood cells to repair the anemia state. That is, the increase in monocytes and new erythropoiesis occur simultaneously in anemia (39). This indicates that anemia affects the number of monocytes. In our study, hemoglobin at diagnosis was lower in the non-cured group than in the cured group, but the difference was not significant (*P* = 0.065). However, monocyte at diagnosis was significantly higher in the non-cured group than in the cured group (*P* < 0.0001). Multivariate logistic regression analysis showed that monocyte remained an independent risk factor for the prognosis of A-TB after adjusting hemoglobin and red blood cells (Supplementary Table 3). To sum up, monocyte may influence the prognosis of A-TB by affecting anemia through other pathways, which provides a new direction for further research to explore the mechanisms.

Cavity has negative impacts on patients and was associated with poor treatment outcomes, including delayed conversion of sputum culture, relapse after treatment, and development of drug resistance (40). Immunocompromised TB patients have a higher prevalence of multiple small cavities within TB lesions compared to patients without underlying disease (41). The previous study has found a higher incidence of cavities, a larger area of cavities, and a longer recovery time from cavities in A-TB, suggesting that anemia caused more serious pathological consequences in TB patients (6). It is suggested that anemia is a risk factor for cavitation in TB patients. Monocytes at baseline affects the prognosis of TB through anemia, which is not only an independent risk factor for A-TB, but could also theoretically influence the changes of cavitation in A-TB. In this study, we further found that patients with monocyte < 0.535 × 10^9/L had a higher rate of cavity closure in A-TB, and multivariate Cox regression model analysis confirmed that monocyte was a biomarker for predicting cavity closure. Monocyte at baseline was not merely an independent predictor of the prognosis of A-TB, but also a biomarker for predicting cavity closure in A-TB.

A number of limitations should be considered when interpreting the results of this analysis. First, the present study was a single-center retrospective study with the inherent weaknesses of a retrospective study. Second, the sample size was relatively small. In addition, the follow-up period of this study was relatively short, and further prospective studies are required to confirm the results of this study and to clarify the mechanism of the effect of peripheral monocytes on clinical outcomes.

In summary, this is the first study to provide evidence showing that monocyte at diagnosis can be used as a biomarker of A-TB prognosis, and it also proves to be an independent predictor of cavity closure in A-TB. Since monocyte at diagnosis is cost-effective and easily measured in clinics and hospitals, it is expected to be extremely useful as a prognostic marker at the time of initial diagnosis. Besides, due to the correlation between immune cells with TB and disease prognosis, promoting basic research on the aspects of immunity, monocyte may lead to the development of new therapies.

## Data availability statement

The raw data supporting the conclusions of this article will be made available by the authors, without undue reservation.

## Ethics statement

Ethical review and approval was not required for the study on human participants in accordance with the local legislation and institutional requirements. Written informed consent from the participants was not required to participate in this study in accordance with the national legislation and the institutional requirements.

## Author contributions

ZL and YH designed and directed the study and assisted in writing the manuscript. ML, XZ, and QZ were responsible for data collection, statistical analysis, data analysis, and manuscript writing. YW and FY contributed to the data collection. HY, LQ, and RZ contributed to the interpretation of the results. All authors contributed to the article, critically read and commented on the manuscript, and approved the submitted version.
