# Supplementary material for: Monocyte at diagnosis as a prognosis biomarker in tuberculosis patients with anemia
Source: Front Med (Lausanne). 2023 Jun 7;10:1141949. doi: 10.3389/fmed.2023.1141949 (PMC10282774; doi:10.3389/fmed.2023.1141949)
Supplement: Supplementary file 1 [file Table_1.docx]

Monocyte at diagnosis as a prognosis biomarker in tuberculosis patients with anemia

patients with anemia

Mengxing Luo^12✝^, Xin Zou^12✝^, Qibing Zeng^1✝^, Yaxing Wu^1^, Hua Yang^2^, Lianhua Qin^2^, Ruijuan Zheng^2^, Fangyong Yu^2^, Yang Hu^2*^, Zhonghua Liu^12*^

^1^The Key Laboratory of Environmental Pollution Monitoring and Disease Control, Ministry of Education, School of public health, Guizhou Medical University, Guiyang, China.

^2^Shanghai Key Laboratory of Tuberculosis, Shanghai Pulmonary Hospital, Tongji University School of Medicine, Shanghai, China.

*** Correspondence:**Yang Hu, Email:huyang3141@163.com

Zhonghua Liu, Email:nllzh@126.com

^✝^Mengxing Luo^12✝^, Xin Zou^12✝^,and Qibing Zeng^1✝^These authors have contributed equally to this work and share first authorship

The number of supplementary tables:3

Supplementary table 1 Drug resistance in 181 A-TB patients

|  | Anemia | |
| --- | --- | --- |
|  | Cured group(n=94) | Non-cured group(n=87) |
| Drug resistance | 2 | 7 |
|  | RR-TB(H) | RR-TB(H) |
|  | MDR-TB(SHR) | RR-TB(H) |
|  |  | RR-TB(R) |
|  |  | RR-TB(R) |
|  |  | RR-TB(HS) |
|  |  | MDR-TB(RH) |
|  |  | MDR-TB(SHREZ) |

Abbreviations:RR-TB, rifampicin-resistant TB; MDR-TB, multidrug-resistant TB;

H, Isoniazid; R, Rifampin; S, Streptomycin; E, Ethambutol; Z, Pyrazinamide.

Supplementary table 2 Univariate analysis of peripheral blood cells of tuberculosis patients without anemia in cured and non-cured groups

| Factor | Non-anemia | | P value |
| --- | --- | --- | --- |
|  | Cured group(n=137) | Non-cured group(n=118) |  |
| MONO,10^9/L | 0.56(0.43-0.69) | 0.52(0.41-0.69) | 0.383 |
| HGB,g/L | 135.84±12.58 | 133.71±12.39 | 0.177 |
| RBC,10^12/L | 4.62±0.39 | 4.61±0.41 | 0.712 |
| NEUT,10^9/L | 3.67(2.90-4.68) | 3.77(2.87-4.47) | 0.806 |
| LYM,10^9/L | 1.81±0.59 | 1.61±0.48 | 0.004 |
| EO,10^9/L | 0.11(0.07-0.18) | 0.11(0.08-0.19) | 0.701 |
| BASO,10^9/L | 0.02(0.01-0.03) | 0.02(0.01-0.023) | 0.041 |
| PLT,10^9/L | 244.29±68.29 | 245.17±62.04 | 0.915 |
| HCT,L/L | 0.41±0.03 | 0.40±0.03 | 0.080 |
| MCV,fL | 90.00(86.00-92.00) | 87.98±5.06 | 0.024 |
| MCH,pg | 30.00(28.00-31.00) | 29.00(28.00-30.00) | 0.068 |
| MCHC,g/L | 329.96±10.82 | 330.69±10.51 | 0.587 |
| PCT,% | 0.25(0.21-0.28) | 0.26±0.06 | 0.431 |
| PDW,% | 12.05(10.70-13.40) | 11.70(10.85-13.45) | 0.883 |
| MPV,fL | 10.45±1.08 | 10.49±1.07 | 0.849 |

No difference in monocyte, red blood cell, platelet and hematocrit were statistically significant in univariate analysis.

P<0.05 is considered statistically significant.

Abbreviations:MONO,monocyte; HGB,hemoglobin; RBC,red blood cell; NEUT,neutrophil; LYM,lymphocyte; EO,eosinophil; BASO,basophil; PLT,platelet; HCT,Hematocrit; MCV,mean corpuscular volume; MCHC,Mean hemoglobin concentration; PCT,platelet crit; PDW,platelet distribution width; MPV,mean platelet volume.

Supplementary table 3 Multivariate logistics analysis of the correlation between MONO and poor prognosis

| Factor | OR(95%CI) | P^a^ Value | OR1(95%CI) | P^b^ Value |
| --- | --- | --- | --- | --- |
| MONO,10^9/L | 10.206(2.451-42.497) | 0.001 | 10.714(2.349-48.872) | 0.002 |
| HGB,g/L | 0.982(0.957-1.009) | 0.187 | 0.984(0.957-1.012) | 0.253 |
| RBC,10^12/L | 0.665(0.300-1.476) | 0.316 | 0.654(0.288-1.487) | 0.311 |

^a^ In multivariate logistics analysis, MONO was a risk factor for poor prognosis.

^b^ After adjusting for age and sex, MONO remained an independent risk factor for poor prognosis.
